# Bridging the Gap between Single Molecule and Ensemble Methods for Measuring Lateral Dynamics in the Plasma Membrane

**DOI:** 10.1371/journal.pone.0078096

**Published:** 2013-12-04

**Authors:** Eva C. Arnspang, Jeremy Schwartzentruber, Mathias P. Clausen, Paul W. Wiseman, B. Christoffer Lagerholm

**Affiliations:** 1 Department of Physics, Chemistry and Pharmacy, MEMPHYS-Center for Biomembrane Physics & DaMBIC – Danish Molecular Biomedical Imaging Center, University of Southern Denmark, Odense, Denmark; 2 Department of Physics and Department of Chemistry, McGill University, Montreal, Canada; Institut Jacque Monod, Centre National de la Recherche Scientifique, France

## Abstract

The lateral dynamics of proteins and lipids in the mammalian plasma membrane are heterogeneous likely reflecting both a complex molecular organization and interactions with other macromolecules that reside outside the plane of the membrane. Several methods are commonly used for characterizing the lateral dynamics of lipids and proteins. These experimental and data analysis methods differ in equipment requirements, labeling complexities, and further oftentimes give different results. It would therefore be very convenient to have a single method that is flexible in the choice of fluorescent label and labeling densities from single molecules to ensemble measurements, that can be performed on a conventional wide-field microscope, and that is suitable for fast and accurate analysis. In this work we show that k-space image correlation spectroscopy (kICS) analysis, a technique which was originally developed for analyzing lateral dynamics in samples that are labeled at high densities, can also be used for fast and accurate analysis of single molecule density data of lipids and proteins labeled with quantum dots (QDs). We have further used kICS to investigate the effect of the label size and by comparing the results for a biotinylated lipid labeled at high densities with Atto647N-strepatvidin (sAv) or sparse densities with sAv-QDs. In this latter case, we see that the recovered diffusion rate is two-fold greater for the same lipid and in the same cell-type when labeled with Atto647N-sAv as compared to sAv-QDs. This data demonstrates that kICS can be used for analysis of single molecule data and furthermore can bridge between samples with a labeling densities ranging from single molecule to ensemble level measurements.

## Introduction

There are several methods for characterizing the lateral dynamics of the molecular organization in the plasma membrane of mammalian cells. These methods include single focus spot measurement techniques such as fluorescence recovery after photobleaching (FRAP) [1], [2], fluorescence correlation spectroscopy (FCS) [2], [3] and STED-FCS [4], multi-spot measurements such as scanning-FCS [3], and fluorescence imaging techniques such as single particle tracking (SPT) [5], and a variety of image correlation spectroscopy (ICS) techniques, e.g. temporal image correlation spectroscopy (TICS) [6], spatio-temporal image correlation spectroscopy (STICS) [7], raster scan image correlation spectroscopy (RICS) [8], [9], k-space image correlation spectroscopy (kICS) [10], and particle image correlation and cross-correlation spectroscopy (PICS, and PICCS) [11], [12].

For example traditional imaging techniques and associated ICS analysis techniques (TICS, STICS, RICS, and kICS)as well as spot measurement techniques (FRAP, FCS, and scanning-FCS) are diffraction-limited and hence restricted to investigate dynamics at length scales greater than about 250 nm. Of these techniques TICS, kICS, FRAP, and the various versions of FCS are furthermore limited to only be able to report on the ensemble average spatial and temporal dynamics, while RICS and STICS can resolve spatial but not temporal heterogeneities. In contrast STED-FCS and SPT are able to sample lateral dynamics at sub-diffraction limited length scales; in the case of STED-FCS down to about 40 nm while the lower length scale for SPT is only dependent on the precision by which single molecules can be localized. SPT is furthermore in the case of the use of photostable probes (e.g. 40 nm Ø gold particles, QDs) able to differentiate between single molecule behaviors. The sub-diffraction limited spatial resolution in SPT and related techniques is, however, only possible in the case of very low labeling densities, <1 one labeled molecule µm^−2^, as compared to the estimated protein density in the plasma membrane of 25,000 proteins µm^−2^ [13]. Hence, there is a possibility that SPT results in the sub-sampling of the lateral dynamics of only a very small distinct population of single molecules that are readily accessible to the particular probe. This problem is expected to be more severe in the case of the use of large probes, as well as, in studies of samples that contain large topological differences, e.g. measurements of lateral dynamics in the microvillus in the brush border of epithelial cells [14], [15]. Use of larger probes furthermore increases the risk for probe induced artifacts due to sterics and cross-linking [15].In the end it is of course important to note that all these methods have distinct advantages and disadvantages. In particular, it is very evident that observed lateral dynamics of lipids and proteins in the plasma membrane of live cells is very dependent on the specifics of the data acquisition as well as the choice of label. It is therefore of upmost importance that methods that can bridge over different spatial and temporal scales and further are label and labeling density independent are further developed.

In the case of SPT, the analysis is also computationally very intensive as it requires extensive non-linear curve fitting of imaged spots in each image frame of a time-lapse sequence to approximations of the microscope point spread function (PSF), e.g. two dimensional spatial Gaussians, and subsequent linking of particle positions into particle trajectories describing the motion of single molecules [16]–[18]. It would therefore be very useful to have a complementary measurement technique to traditional SPT analysis that can be applied to the same data, that is computationally less intensive and thus faster to perform, that yields comparable accurate results of the observed single molecule dynamics.

This has previously been addressed by the introduction of particle image correlation spectroscopy (PICS) analysis from which diffusion coefficients can be determined from datasets in which single particles are resolved [11]. PICS analysis has been shown to give the same results as SPT to also include information about molecular spatial confinement [11]. In this work we show that k-space image correlation spectroscopy (kICS) like PICS can also be used to analyze single particle tracking data accurately. kICS is a recently developed image correlation spectroscopy technique that time correlates the Fourier (k)-space representation of images, and thereby separates contributions due to time dependent photophysical fluctuations from those due to transport in and out of the PSF [10].Further, kICS analysis in contrast to PICS does not require individual single particle localizations. In fact kICS analysis can be applied to datasets independently of the labeling densities [10]. However, while PICS can also obtain information about spatial confinement, kICS cannot.

kICS has previously been applied to single molecule density data of QD-labeled membrane proteins [19], but until now no direct comparison had been made with SPT data. In order to further validate the use of kICS analysis for SPT data we here report on the direct quantitative comparison of diffusion coefficients retrieved using kICS for two datasets of QD-labeled plasma membrane components with those from SPT analysis. We find the results to be in good agreement. We have further used kICS to investigate the effect of the label size by comparing the diffusion coefficients retrieved when labeling an artificially biotinylated lipid (biotin-cap-DPPE) with streptavidin (sAv) conjugated QDs as opposed to sAv that was labeled with the fluorescent dye Atto647N. In this latter case, the labeling was further done at high labeling densities hence illustrating that kICS analysis can effectively bridge the gap between single molecule analysis and ensemble average analysis methods. In this latter comparison, we further found that the diffusion coefficient for the lipid is two-fold greater when labeling was by sAv-Atto647N rather than by commercially available sAv-QDs.

## Results and Discussion

### Multi-color QD Labeling and Imaging

**Figure 1:**
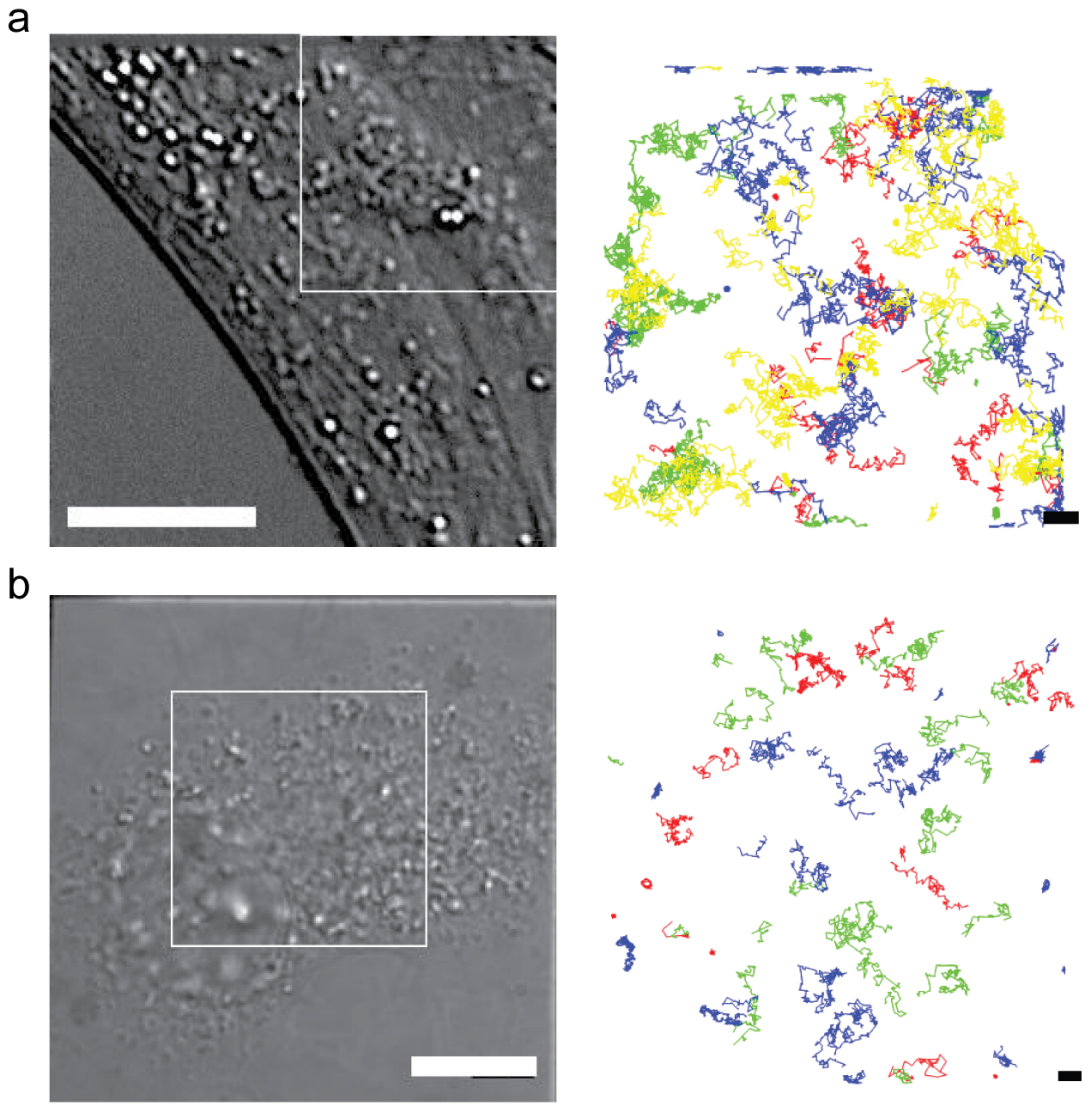
Multi-color QD single molecule imaging in live MEFs. Differential interference contrast images (left column, scale bar = 10 µm) and superimposed trajectories from SPT analysis (right column, scale bar = 1 µm) in selected regions-of-interests (ROIs). (a) Four color single QD time-lapse imaging of an artificial lipid, biotin-cap-DPPE, labeled by a four color combination of sAv-QD705 (red), sAv-QD655 (green), sAv-QD605 (blue), and sAv-QD565 (yellow). (b) Three color single orthogonal QD imaging of the sphingolipid, G_M1_, with ChToxB-QD705 conjugates (red), ACP-CD59with CoA-QD655 conjugates (green), and BLAP-EGFR with sAv-QD605 conjugates (blue).

Representative images of each of the two biological systems and regions of interests (ROIs) that were analyzed in this work are shown in Figure 1. The image acquisition rate, f, in both examples was 25 Hz resulting in an acquisition time interval, τ, of 1/f = 40 ms. These studies were performed in a clonal mouse embryonic fibroblast (MEF) cell line (IA32) from a knockout mouse for the INK4a/ARF (−/−) locus [20]–[22]. This locus encodes two tumor suppressor genes, p16(INK4a)and p19(ARF), which both play an important role in cancer [23]. This clonal cell line was chosen for this work because they are atypically large and flat which makes them ideal to use for SPT measurements and the subsequent 2D analysis that was used in this work. In the first example, we have imaged the lateral dynamics of artificially loaded lipids, biotin-cap-DPPE, in a live mouse embryo fibroblast (MEF). This data-set was previously presented in its entirety in [22]. For the purpose of this work, we have re-analyzed two ROIs from this dataset. The artificially loaded lipids were labeled with a four color combination of sAv-QDs, with reported fluorescence peak emission of 565, 605, 655, and 705 nm, respectively, and the lateral dynamics was imaged simultaneously with 5 ms integration. With this color combination of QDs, and with the described filter combination in the Materials and Methods section, there is minimal bleed through of fluorescence from one channel to the next [22]. In the second biological system, we have simultaneously imaged (with 10 ms camera integration) the lateral dynamics of a sphingolipid, G_M1_, with ChToxB-QD705 conjugates, an ACP-CD59 with CoA-QD655 conjugates and BLAP-EGFR with sAv-QD605 conjugates and analyzed two ROIs from one dataset, hence showing that orthogonal simultaneous multi-color single QD imaging is also possible. In both examples, we have further minimized the impact of QD photobleaching and intermittency by addition of 25–50 µM β-mercaptoethanol (β-ME) in the imaging media [24]. Addition of β-ME at these concentrations is physiologically permissible; in fact human stem cells are generally grown in 100 µM β-ME [25] and 50 µM β-ME has been shown to promote proliferation of human osteoprogenitor cells [26]. The addition of up to 50 µM β-ME has also previously been shown have no effect on the magnitude of the diffusion coefficient of BLAP-EGFR in a live MEF.

### kICS Analysis of Single QD Imaging Data

Prior to analysis of experimental single molecule data by kICS, we investigated whether the molecular number densities had an effect on the extracted average diffusion coefficient, 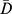. In this case, we analyzed simulated data at varying particle densities from 0.01 to 1000 particles/µm^2^. This analysis showed that the magnitude of the fitted diffusion coefficient, 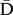, from simulated data was independent of the particle density (data not shown).

**Figure 2:**
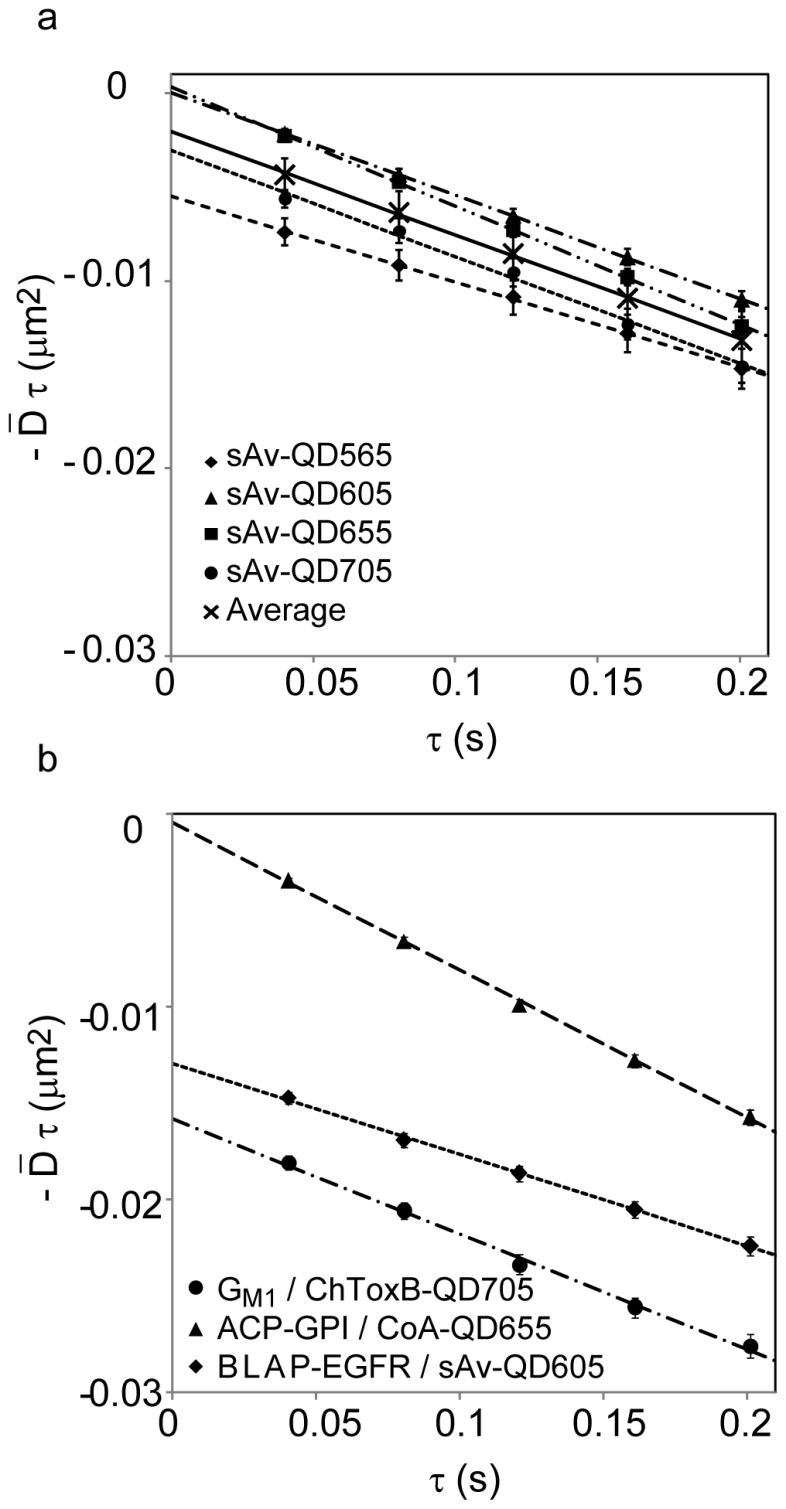
Analysis of lateral dynamics by kICS. The slopes of each average correlation function ln (r(**k** ^2^,τ))from two ROIs at different τ values are plotted as a function of τ. The slope of each plot gives the diffusion coefficients listed in Tables 1 and 2. (a) Four color single QD time-lapse imaging of an artificial lipid, biotin-cap-DPPE, labeled by a four color combination of sAv-QDs. (b) Three color single orthogonal QD imaging of the sphingolipid, G_M1_, with ChToxB-QD705s, ACP-CD59 with CoA-QD655s, and BLAP-EGFR with sAv-QD605s.

kICS analysis was subsequently performed in two ROIs each for the two experimental data sets (Figure 1). In all cases, the correlation decays were fit to image frame lags, 1≤n τ≤5, corresponding to time lags 40≤n τ≤200 ms. The correlation decay plots were linear for all molecules and labels suggesting that the particles on average follow free diffusion over this time interval and at these sampling intervals [10]. Representative examples of the analysis for each data set and one ROI are shown in Figure 2. The magnitudes of the average diffusion coefficients, 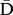 from both ROIs for each label condition in the case of the biotin-cap-DPPE lipid labeled with sAv-QD565, sAv-QD605, sAv-QD655 and sAv-QD705 are given in column 1 of Table 1. The average diffusion coefficients, 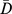, from both ROIs for each label condition in the case of orthogonal labeling of BLAP-EGFR labeled with sAv-QD605, ACP-CD59 labeled with CoA-QD655 and G_M1_ labeled with ChToxB-QD705 are given in column 1 of Table 2.

**Table 1:** Quantitative comparison of kICSand SPT analysis for four QD color set.

	kICS Analysis (40≤n τ≤200 ms)	SPT Average Trajectory Analysis (40≤n τ≤200 ms)	SPT Single Trajectory Analysis (40≤n τ≤200 ms)
	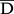 ± ASE(µm^2^/s)	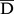 ± ASE(µm^2^/s)	*D* ± STD (µm^2^/s)
Biotin-cap-DPPE/sAv-QD565	0.045±0.004	0.054±0.001	0.055±0.020 (N_Traj_ = 122)
Biotin-cap-DPPE/sAv-QD605	0.055±0.000	0.061±0.003	0.062±0.025 (N_Traj_ = 69)
Biotin-cap-DPPE/sAv-QD655	0.064±0.001	0.050±0.001	0.055±0.021 (N_Traj_ = 48)
Biotin-cap-DPPE/sAv-QD705	0.057±0.002	0.055±0.001	0.055±0.017 (N_Traj_ = 63)

**Table 2:** Quantitative comparison of kICS and SPT analysis for three QD color set.

	kICS Analysis (40≤n τ≤200 ms)	SPT Average Trajectory Analysis (40≤n τ≤200 ms)	SPT Single Trajectory Analysis (40≤n τ≤200 ms)
	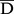 ± ASE(µm^2^/s)	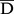 ± ASE(µm^2^/s)	*D* ± STD (µm^2^/s)
BLAP-EGFR/sAv-QD605	0.047±0.010	0.074±0.003	0.070±0.048 (N_Traj_ = 40)
ACP-CD59/CoA-QD655	0.076±0.007	0.103±0.002	0.100±0.039 (N_Traj_ = 51)
G_M1_/ChToxB-QD705	0.060±0.011	0.060±0.003	0.057±0.064 (N_Traj_ = 22)

### SPT Analysis of Single QD Imaging Data

The same ROIs and image frames analyzed by kICS were also analyzed by SPT analysis as described in the Materials and Methods. Worth noting is that single molecule imaging with QDs, as a result of QD intermittency, in general results in the detection of many short trajectories, rather than a few long trajectories. For example, the results for the number of detected trajectories, N_Traj_, and statistics of the number of displacements, n, of the detected trajectories in the case of the single molecule analysis of the lipid, biotin-cap-DPPE, that was labeled with sAv-QD565 and sAv-QD705 for a specific ROI, are given in Table 3. These numbers are representative of all ROIs and for all QD colors that were analyzed as part of this work.

**Table 3:** Example of the results of the QD trajectory linking.

	sAv-QD565		sAv-QD705	
	Primary Linking	Secondary Linking (n>20)	Primary Linking	Secondary Linking (n>20)
N_Traj_	1501	112	1013	76
∑N_Displ_,	14,275	9,815	7,345	4,450
N_Displ_^Max^	547	553	238	817
N_Displ_^Min^	1	21	1	21
N_Displ_^Med^	2	52	3	36
N_Displ_^Mean^	10±31	88±94	7±15	59±95

**Figure 3:**
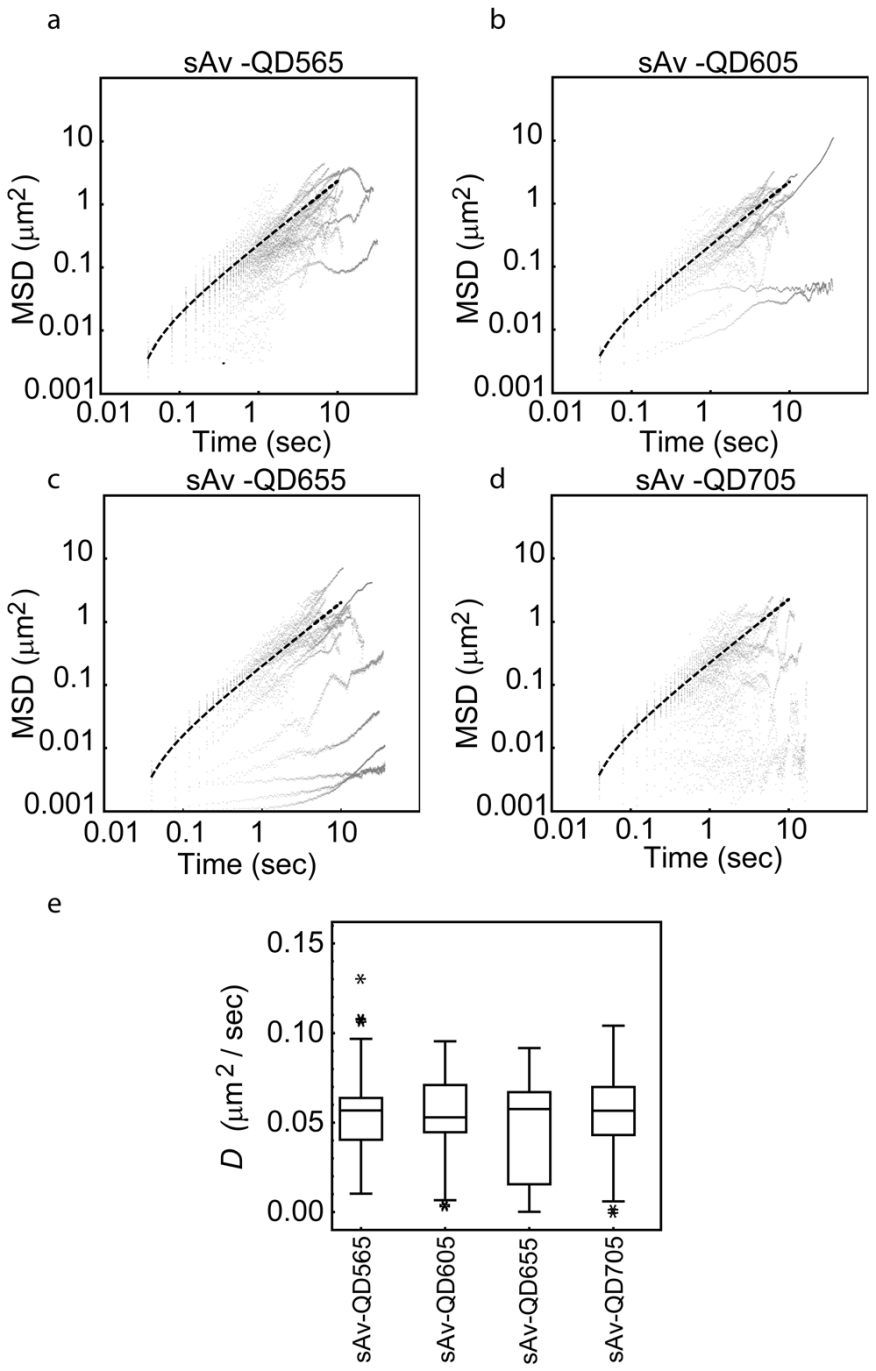
SPT analysis of dynamics of biotin-cap-DPPE labeled with a four color combination of sAv-QDs. Shown are the results of the time averaged single molecule analysis of the same sub-region as the for the kICS analysis in Figure 2a. (a–d)Log_10_ (MSD) versus log_10_ (τ) plots for all detected single molecule trajectories, N_Traj_, with length, n>20 image frames, in each separate QD color channel describing the motion of a lipid, biotin-cap-DPPE, labeled with sAv-QDs in a live MEF. Also shown in each plot is the linear fit of the average MSD of all trajectories at short time lags, 1≤n≤5 corresponding to 40≤n τ≤200 ms, to Eq. 6. The resulting fit values for the time averaged diffusion coefficients, 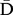,for this labeling example is shown in Table 2. (e) Box-and-whisker representation of the fitted diffusion coefficients, D, from all detected single molecule trajectories, N_Traj_, with length, n>20 image frames and that were statistically best described by the free diffusion model.

**Figure 4:**
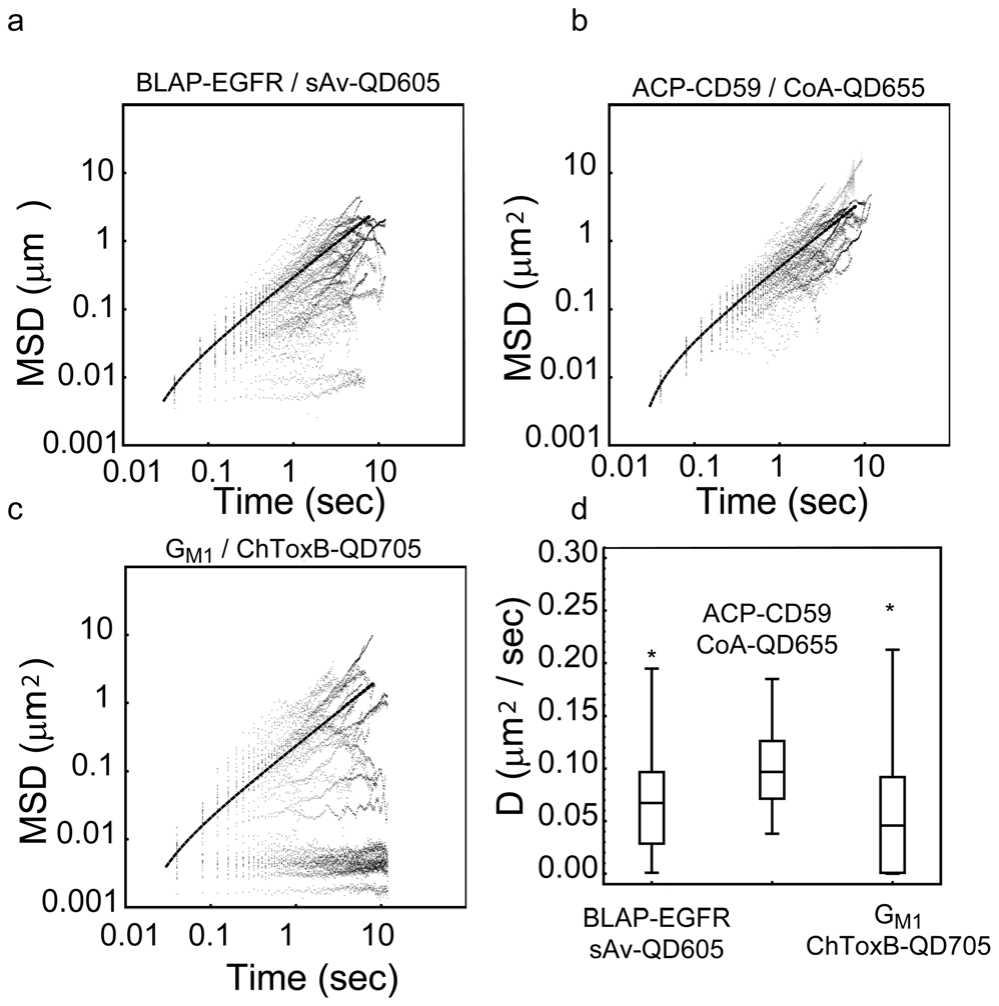
SPT analysis of simultaneous for three color orthogonal QD labeling in live MEFs. Shown are the results of the time averaged single molecule analysis of the same sub-region as the kICS analysis in Figure 2b. Log_10_ (MSD) versus log_10_ (τ) plots for all detected single molecule trajectories, N_Traj_, with length, n>50 image frames, in each separate QD color channel describing the motion of (a) G_M1_ labeled with ChToxB-QD705s, (b) ACP-CD59 labeled with CoA-QD655s, and (c) BLAP-EGFR labeled with sAv-QD605s. Also shown in each plot is the linear fit to Eq. 6 of the average MSD of all trajectories at short time intervals, 1≤n≤5 corresponding to 40≤n τ≤200 ms. (d) Box-and-whisker representation of the fitted diffusion coefficients, D, from all detected single molecule trajectories, N_Traj_, with length, n>50 image frames and that were statistically best described by the free diffusion model.

To be able to compare the results of the SPT analysis to the kICS analysis, we first analyzed the time average of the mean squared displacement for each QD color, in each ROI, in each data set. In this case, we first calculated the mean squared displacement (MSD) for all single trajectories, with a length of n>20 displacements, for each QD label condition, for all possible time lags, n τ. We next calculated the average MSD from all trajectories for a given label condition for very short image frame lags, 1≤n≤5, corresponding to 40≤n τ≤200 ms (Eq. 5), and curve fit the result to the expression for free diffusion (Eq. 6). Representative examples of the results of this analysis for each biological system and for all possible lipid∶QD and membrane protein∶QD combinations are shown in Figures 3 and 4. The magnitudes of the average diffusion coefficients, 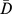, from both ROIs for each label condition as determined by SPT analysis in the case of the biotin-cap-DPPE lipid labeled with sAv-QD565, sAv-QD605, sAv-QD655 and sAv-QD705 are given in column 2 of Table 1. The magnitudes of the average diffusion coefficients, 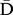, from both ROIs for each label condition as determined by SPT analysis in the case of orthogonal labeling of BLAP-EGFR labeled with sAv-QD605, ACP-CD59 labeled with CoA-QD655 and G_M1_ labeled with ChToxB-QD705 are given in column 2 of Table 2.

We furthermore performed a single trajectory analysis of trajectories with n>50 displacements by curve fitting to the MSD for each separate trajectory, at short time intervals, 40≤n τ≤200 ms, to three nested diffusion models, free diffusion (Eq. 7), confined diffusion (Eq. 8), and a combination of free and confined diffusion (Eq. 9). In this case we further used simulations of free diffusion in an infinite 2D-plane, in order to determine the accuracy of the single trajectory analysis of trajectories with a length of n = 50. These simulations showed that we could recover the magnitude of the simulated diffusion coefficient at short time intervals within a 20% accuracy (data not shown). Importantly, single trajectory analysis makes it possible for that the motion of each single molecule can be classified by use of e.g. F-statistics (Eq. 11), in order to statistically determine the simplest model that described the motion of each separate molecule. In this analysis, we found that a large majority of all single trajectories, for the chosen short time interval, and for all possible combinations of labeled lipids and proteins were statistically best described by a simple free diffusion model (Eq. 7). In the case of all analyzed single trajectories of biotin-cap-DPPE labeled by either sAv-QD565, sAv-QD605, sAv-QD655, or sAv-QD705, this was true for 302 out of a total of 316 analyzed trajectories (302/316). Similar results were found in the case of ACP-CD59 labeled with CoA-QD655 where 51/52 trajectories were best described as free diffusion; while in the cases of G_M1_ and BLAP-EGFR, 22/33, respectively 40/51 analyzed trajectories were best described by free diffusion. These results also supports our assumption that the investigated membrane molecules on average follow free diffusion over the analyzed short time interval, 40≤n τ≤200 ms, and at the sampling intervals of τ = 40 ms that were used in these experiments.

The fitted magnitudes of the diffusion coefficients, D, for all trajectories that were statistically best described by a free diffusion model (Eq. 7) are presented in the form of Box- and- Whisker in Fig. 3e for the case of the biotin-cap-DPPE lipid labeled with a four color combination of sAv-QDs, and in Fig. 4d for the case of orthogonal QD labeling of BLAP-EGFR, ACP-CD59, and G_M1_. These plots show that even though a majority of trajectories are best described as free Brownian diffusion, there is a large heterogeneity in the magnitude of the fitted diffusion coefficients, D, in all instances with examples ranging from immobile molecules (D<0.001 µm^2^/s) to fast moving molecules (D≥0.1 µm^2^/s). This heterogeneity is not unexpected and is for example also consistent with the presence of an immobile fraction in FRAP data [2] or the presence of multiple components in cumulative diffusion analysis [27]. It can, however, be seen that the average of the diffusion coefficient, D, from the single trajectory analysis is in all instances in very close agreement with the average diffusion coefficient, 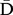 from the average SPT analysis. This then provides a validation that an average SPT analysis, at least for the short term time window of 40≤n τ≤200 ms that was analyzed here, is sufficient to determine the magnitude of the average diffusion coefficient. This is gratifying result as in the case of SPT with fluorescent dyes and proteins an average SPT analysis is typically also the only option as a result of the collection of very short trajectories (typically n<<50) due to photobleaching [15], [27]

### Comparison of SPT and kICS Analysis

The magnitudes of the average diffusion constants, 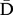, for both types of analysis are listed in columns 1 and 2 of Tables 1 and 2. From these results in can be seen that the average magnitude of the diffusion coefficient, 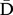, for all molecules at the investigated image frame lags, 1≤n≤5, and corresponding time lags 40≤n τ≤200 ms are in the range of 0.05–0.10 µm^2^/s. From these results it can be seen that the results from the two types of analysis are in agreement (within 25%) in the case of the data set of the lateral dynamics of biotin-cap-DPPE labeled by either sAv-QD color conjugate. The discrepancy, however, is much larger in the case of the second data set where the disagreement is >30% in the case of ACP-CD59 labeled with CoA-QD655 and for BLAP-EGFR labeled with sAv-QD605 while there is perfect agreement for the case of GM1 labeled by ChToxB-QD705. From the single trajectory analysis results in column 3 of Tables 1 and 2 (and the Box- and Whisker plots in Fig. 3e and 4d), it is also very apparent that the magnitude of the average diffusion coefficients, 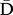, as determined by either kICS or by SPT averages over significant heterogeneity among the observed single molecule motion ranging from immobile molecules (D<0.001 µm^2^/s) to fast moving molecules (D≥0.1 µm^2^/s). The large heterogeneity in the single molecule motion is further apparent from the representative trajectories in Figure 1. From the analyzed examples it can also be seen that the heterogeneity is much greater in the case of the second example (Figure 1b) where the discrepancy between the kICS and the average SPT analysis was greater. Importantly though it can further be seen that the results for the magnitude of the average diffusion coefficients, 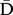, from kICS and SPT in all instances fall within one standard deviation of the magnitude of the diffusion coefficients, D, from the single trajectory SPT analysis.

There are several possible reasons for the observed discrepancies. In the kICS analysis the contributions to the temporal correlations from background fluorescence and non-moving molecules are removed by a pre-normalization step in Fourier space. Subsequently, the weight given to a molecule depends on the square of its fluorescence intensity, since the correlation is a product of two k-space images. In contrast, in the average SPT analysis all the trajectories are first identified and the MSD curves are calculated for each single trajectory separately. In this analysis, the identified trajectories are independent of particle brightness, provided that a given particle can in fact be tracked, and furthermore that the resulting trajectories contain, n>20 displacements. In this analysis, we furthermore include all trajectories independent of the extent of the particle mobility. The average MSD curve for each labeled molecule is subsequently calculated by placing equal weights per trajectory resulting in that non-moving and moving molecules are weighted equally. These differences in the treatment of the measured molecule displacements in the two analysis methods result indifferences in the fitted average diffusion coefficient, 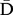 even though the same data sets are analyzed. The indication from the data sets that were analyzed here is further that the discrepancy increases as the heterogeneity in the single molecule movement increases.

### kICS analysis of High Density Labeling Single-Color Imaging Data

A very important advantage of the kICS analysis is that in contrast to SPT and PICS analysis which requires very sparse labeling densities, kICS can also be used for quantifying lateral dynamics at high labeling densities [10]. In this work, we initially tried to take advantage of this by investigating the effect of the labeling density on the measured diffusion rates. Initially, we made several unsuccessful attempts to increase the labeling density of artificially loaded lipids labeled with sAv-QDs such that SPT analysis could not be performed (data not shown). In contrast using similar labeling conditions, high density bulk labeling was readily achieved of artificially loaded lipids when labeling was by Atto647N labeled sAv. This further suggests that a limitation of single molecule microscopy with bulky probes (e.g. QDs and gold particles) is that it is very likely that only a subset of the molecules of interest are accessible for labeling by these bulky probes.

**Figure 5:**
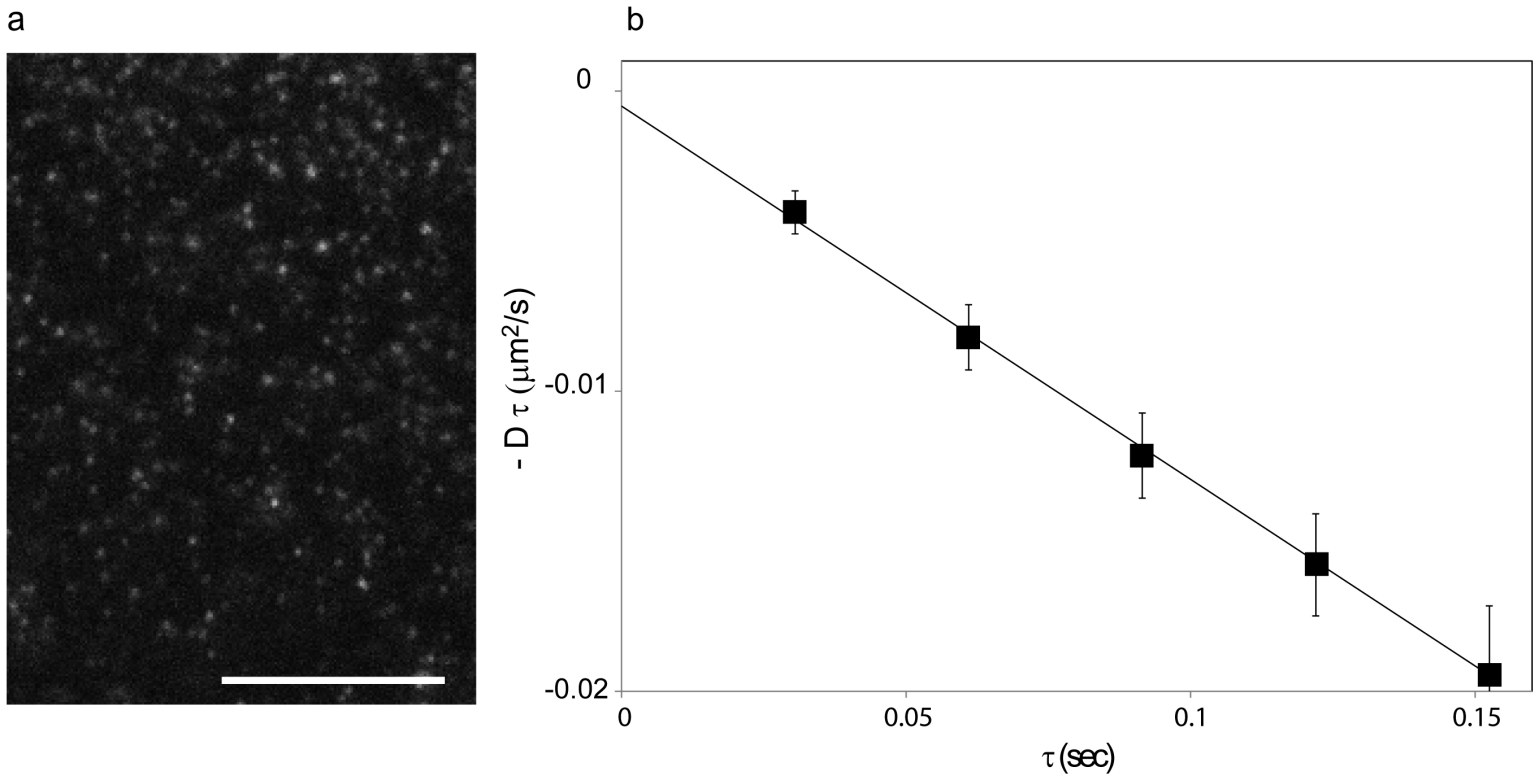
High density labeling single-color imaging of single molecules in live cells. (a) One ROI of a 1200 frame image sequence of DPPE-biotin-Atto647N-sAv labeled MEF. (Scale bar = 10 µm) Fluorescence images were acquired at 30 Hz. Resulting fluorescence images were analyzed as described in Materials and Methods. In this example, we analyzed one ROI from each of 5 datasets of 600 image frames that were acquired with 30 ms integration. The average diffusion coefficient of biotin-cap DPPE was quantified by kICS analysis to be 0.12±0.01 µm^2^/s. (b) Analysis of lateral dynamics by kICS. Single color time-lapse imaging of an artificial lipid, biotin-cap-DPPE, labeled by Atto647N-sAv. The slope of the average correlation function ln (r(**k** ^2^,τ))from 5 datasets at different τ values are plotted as a function of τ.

We were also interested in determining the effect that the bulky QDs have on the measured diffusion rates. Previous studies have for example shown that the measured diffusion rates of membrane molecules, for a similar time window as what we used here, that were labeled with fluorescent dyes was about twice as fast as compared to molecules that were labeled with large gold particles [28]. For this comparison, we used kICS analysis to investigate the impact that the size of the external label has on the diffusion rate. In this case we used kICS to analyze the diffusion rate of the same artificially loaded biotin-cap-DPPE that was used before but that was labeled with Atto647N-sAv (as opposed to sAv-QDs) at bulk densities. The imaging in this case was performed with 30 ms integration and an acquisition rate, f, of 30 Hz by use of an Andor EMCCD camera (Figure 5a). The high labeling density in this case precluded SPT analysis but using kICS analysis we obtained an average diffusion coefficient, 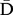, over 5 cells of biotin-cap DPPE of 0.12±0.01 µm^2^/s (Figure 5b). These results are consistent with previous results which have found slower diffusion coefficients for lipids and protein that had been labeled with large gold particles than the same molecules that were labeled with fluorescent dyes [28]. The reasons for this result in this case is not known however there are at least two possibilities for this discrepancy, 1) that the greater size of the QDs affects the dynamics as a result of increased steric interactions, and 2) that the greater paucivalency of the QD probes induces cross-linking.

### Conclusion

In this work we show that k-space image correlation spectroscopy (kICS) analysis can be readily used to accurately analyze the lateral dynamics of sparsely labeled lipids and proteins in the plasma membrane of live cells. Specifically, we show that kICS and traditional SPT analysis of lipids and proteins that had been labeled with QDs yield comparative quantitative results where the results from kICS for the average diffusion coefficients, 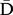, of lipids and membrane proteins in live cells are in all instances within one standard deviation of the average diffusion coefficient as determined by SPT single trajectory analysis. It has previously been shown that another image correlation analysis method, PICS, can also be applied to single molecule data [11] but kICS in contrast to PICS does not require that single molecule localizations and is hence computationally simpler. kICS can furthermore be used to extract diffusion rates from time-lapse image sequences that were labeled at very high labeling densities such that single molecule localization cannot be made.

We have in this work further taken advantage of this ability of kICS in order to investigate the effect of the label size and the labeling density in the case of the lateral dynamics of an artificial biotinylated lipid. In this case we find that the lateral dynamics of the same lipid, in the same cell type, and for the same time-regime is about two-fold faster upon labeling with sAv-Atto647N as compared to either color of sAv-QDs. The exact nature for this discrepancy is not known, however, this effect is consistent with previously published results that compared the lateral dynamics of molecules labeled by either gold particles or fluorescent dyes [28]. Collectively, the data presented here demonstrates that kICS analysis can be used to bridge experiments and experimental results that are performed at a range of labeling densities from single molecules to bulk hence enabling direct comparisons as is the case here of possible artifacts resulting from sparse labeling with larger paucivalent probes as compared to smaller, less invasive probes.

These studies were performed in a clonal mouse embryonic fibroblast (MEF) cell line (IA32) from a knockout mouse for the INK4a/ARF (−/−) locus [20]–[22]. This clonal cell line was chosen for this work because they are atypically large and flat which makes them ideal to use for SPT measurements and the subsequent 2D analysis that was used in this work. Furthermore, a recent study has showed that these cells are able to proliferate in culture after viral (−/−) knock-down of the Arp2/3 complex, a major regulator of the cortical actin cytoskeleton [21]. This makes these cells a very promising model system for investigating the role of the cortical actin cytoskeleton upon the lateral dynamics of lipids and proteins in the plasma. We have previously also shown by SPT studies that were performed at a much higher temporal sampling of 1.7–1.9 kHz than here that a phospholipid (DPPE), a GPI-anchored protein (CD59), and a transmembrane receptor (EGFR) in the plasma membrane of these cells are spatially and temporally transiently confined to nano-compartments with a mean size of about (100 nm)^2^ for a mean period of about 100 ms [29]. Such transient confinement, however, cannot be detected at the very slow temporal sampling rate of 25 Hz that was used in this study. Rather the results obtained at the slow sampling rate of 25 Hz in this case suggest that the lateral dynamics of all molecules that were investigated was best described by a model for simple Brownian diffusion with diffusion coefficients in the range of about 0.05–0.10 µm^2^/s. These results are also consistent with previous studies of the lateral dynamics of the same molecules, e.g. EGFR [30] and CD59 [31], in the plasma membrane of other cell types and for which a similar data acquisition rate was used.

## Materials and Methods

### Cell Culture

MEF cells were grown in a humidified atmosphere at 37°C in 5% CO_2_. Cells were grown until 80–90% of confluence and split every third day in 1∶5–1∶10 ratios using the endo-peptidase Trypsin (Sigma). Cells were grown in Dulbecco's modified eagle's medium with high glucose (DMEM), with standard concentrations of glutamax (Gibco) and penicillin-streptomycin (Sigma) and 10% fetal bovine serum (FBS) (Sigma). For transfection and imaging, cells were seeded on # 1 ½ glass coverslips in 6-well plates at a density of 25,000 cells per well and were transfected at 24 h and imaged within the next 24–48 hours.

### Quantum dot labeling

#### Four color QD labeling

Four color QD labeling was done as previously described [22]. In brief, cells were washed 3× in Dulbecco's PBS with 0.1 g/L CaCl_2_ and 0.1 g/L MgCl_2_(D-PBS) and then loadedwith1,2-dipalmitoyl-*sn*-glycero-3-phosphoethanolamine-N-(cap biotinyl)(biotin-cap-DPPE, Avanti Polar Lipids) by use of fatty-acid free BSA (Sigma, A-8806) [32]. For this labeling, we prepared a 100 µg/ml lipid stock solution in absolute ethanol (stored at 4°C). Cells were then loaded by diluting the lipid stock to 1 µg/ml in 0.1% fatty-acid free BSA in D-PBS and by incubation for 5 minutes at RT. Lipid loaded cells were washed 3× in D-PBS and blocked for non-specific binding in D-PBS with 1% BSA for 1–2 minutes. Cells were subsequently labeled with a four color combination of streptavidin (sAv)-conjugated QDs (Invitrogen) with peak emissions at 565, 605, 655, and 705 nm, respectively. For this labeling, we diluted QD stock solutions to 1 nM each in D-PBS with 1% BSA and filtered the solution with a 0.22 µm syringe filter. Cells were then labeled in 0.5 ml of the QD labeling solution for 2 minutes at RT after which binding was blocked by addition of 100 µl of 1 mM biotin and a further incubation at RT for 2 minutes. Labeled cells were finally washed 3× in D-PBS and imaged in D-PBS with 1% BSA and 50 µM β-mercaptoethanol (β-ME) to minimize QD blinking and spectral shifting [33].

#### Three QD color labeling

Orthogonal three QD color labeling was done as previously described. In brief, expression of the fusion proteins biotin ligase acceptor peptide- epidermal growth factor receptor (BLAP-EGFR) [34] and acyl carrier protein - glucosylphosphatidylinositol anchored protein (ACP-CD59)(New England Biolabs, Ipswich, MA) in MEF cells was done by transient co-transfection of four DNA plasmids, BirA-KDEL (1 µg), BLAP-EGFR (1 µg), ACP-CD59 (1 µg), and a plasma membrane fluorescent protein marker, K-Ras2-YFP (0.25 µg, ATCC plasmid 10089283) per 25,000 cells. Transfections were done using JetPEI (Polyplus transfection) at the recommended 2∶1 (v/w) ratio to the DNA. Transfected cells were grown overnight in 10 µM biotin to enable specific biotinylation of BLAP-EGFR by co-expressed BirA-KDEL plasmid as has been described [35]. The QD labeling was subsequently performed in two steps. First, we labeled the ACP-CD59 fusion protein with custom conjugated coenzyme A (CoA) QDs (peak emission at 655 nm) by washing the cells 3× in D-PBS. Cells were then labeled in 300 µL of filtered (0.22 µm syringe filter) labeling solution prepared with 1 nM of CoA-QDs, 10 mM MgCl_2_, and 0.4 µM ACP-Synthase in DMEM with 10% FBS for 15 minutes at RT. Then labeled cells were washed 3× in D-PBS and blocked in D-PBS with 1% BSA for 1–2 minutes. Cells were then simultaneously labeled with filtered stock solution composed of 200 pM custom conjugated cholera toxin subunit B (ChToxB) QD705s [36] and 1 nM commercial sAv-QD605s (Invitrogen) in D-PBS with 1% BSA for 2 minutes at RT, and blocked with addition of 100 µl of 1 mM biotin and a further incubation at RT for 2 minutes. Labeled cells were finally washed 3× in D-PBS and imaged in D-PBS with 1% BSA and 25 µM β-ME.

#### Atto647N-sAv labeling

Cells were washed 3× in Dulbecco's PBS with 0.1 g/L CaCl_2_ and 0.1 g/L MgCl_2_(D-PBS) and then loaded with biotin-cap-DPPE by use of fatty-acid free BSA (Sigma, A-8806) [32]. For this labeling, we prepared a 100 µg/ml lipid stock solution in absolute ethanol (stored at 4°C). Cells were then loaded by diluting the lipid stock to 5 µg/ml in 0.1% fatty-acid free BSA in D-PBS and by incubation for 5 minutes at RT. Lipid loaded cells were washed 3× in D-PBS and blocked for non-specific binding in D-PBS with 1% BSA for 1–2 minutes. Cells were subsequently labeled with 0.2 µM Atto647N-sAv in D-PBS with 1% BSA. Cells were the labeled for 2 minutes at RT after which binding was blocked by addition of 100 µl of 1 mM biotin and a further incubation at RT for 2 minutes. Labeled cells were finally washed 3× in D-PBS and imaged in D-PBS with 1% BSA.

### Imaging

The QD tracking imaging was done as previously described [22]. In brief, imaging was done on an Olympus IX-81 inverted microscope by use of a 100 W Hg arc lamp for excitation, and a QuadView (MAG Biosystem) image splitter and an EMCCD (Andor, DV887-ECS) for simultaneous excitation and detection of either 3 colors of QDs and YFP (100 X, 1.3 NA oil objective) or 4 colors of QDs (150 X, 1.45 NA oil objective). To accomplish simultaneous multi-color imaging, we used a combination of a 470/40 nm excitation bandpass filter, a Q495LP dichroic filter, and a HQ510LP emission filter (Chroma Technology) all placed in a filter cube in the microscope. The emission was subsequently split into four separate color channels in the QuadView image splitter by use of dichroics at 585, 630, and 690 nm, and emission filters atHQ535/30 m (for YFP) or D565/40 m (for 565 nm QDs), D605/40 m (for 605 nm QDs), D655/20 m (for 655 nm QDs) and an empty position for 705 nm QDs. Using this system, fluorescence time-lapse sequences of labeled cells were acquired at 5 ms (4 color imaging set) or 10 ms (3 color imaging set)integration time for 600–1200 images frames at 25 Hz at RT. In all instances, the acquired image sequences were subsequently split into quadrants resulting in a separate image sequence for each fluorescence channel. These separate fluorescence channels were subsequently manually superimposed by using a differential interference contrast (DIC) image acquired under similar conditions as a reference.

In the case of (DPPE-biotin)-(Atto647N-sAv) labeled cells the imaging was done on an Olympus IX-81 inverted microscope operated in total internal reflection mode using a 640 nm laser for excitation, and an EMCCD (Andor, DV887-ECS) through a 150 X, 1.45 NA oil objective. Using this system, fluorescence time-lapse sequences of the basal membrane of labeled cells at RT were acquired with a camera integration time of 30 ms at a frequency of 25 Hz for 1200 images frames.

### kICS Analysis

kICS analysis was performed as has previously been described [10]. In brief, this analysis was performed separately in each fluorescence time series channel in selected regions-of-interests (ROIs) from an image which had the highest signal-to-noise and the greatest number of QDs. Cropped image series were subsequently Fourier filtered to remove contributions from immobile QDs and the mean background intensity and the k-space time correlation function was calculated from a time stack of 2D spatial Fourier transformed images:
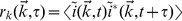
(1)Where 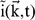 is the Fourier transform of the image acquired at time t, 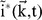 is its complex conjugate, τ is the temporal lag, and the angular brackets denote temporal averaging. For a system with a single component undergoing 2D diffusion, the kICS correlation function has the following form:
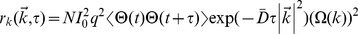
(2)where 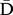 is the average diffusion coefficient, *q* is the quantum yield, *N* is the number of particles in the image, *I*_0_ is the peak incident laser intensity, and *Ω(k)* is the optical transfer function of the imaging system. The photophysical correlation function, *Θ(t) = 1* if fluorophore is on at time t, *Θ(t) = 0* otherwise tracks the photophysics of the fluorophore and is assumed to be independent of particle transport. To obtain a correlation function independent of the point spread function of the imaging system, we divide 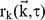 by 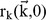 and log-transform:
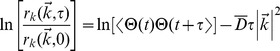
(3)


For each fluorescent species (i.e. each color channel series) we determined 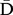 as follows; first, 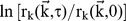 was circularly averaged over 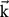, and for each value of τ was plotted in a linear regression as a function of 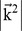. These 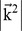 plots were examined to determine cutoff values of 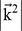 and τ above which noise overwhelmed the correlation signal. Each 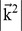 plot has a slope of 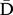
τ, and a linear regression of these slopes as a function of n τ for 1≤n≤5 gave a slope of 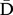.

### SPT Analysis

Similarly to the kICS analysis, the SPT analysis was performed separately for each color channel using the Particle Tracker plug-in in ImageJ [17] as has been described previously [22]. This analysis generates a text file containing the positions of the detected QD particle positions in each image frame as well as linked trajectories describing the motion of individual QDs in time. In this analysis, a major limitation to the use of QDs in SPT is apparent by the generation of a large number of short trajectories rather than a more desirable few very long continuous particle trajectories. In order to minimize the number of inaccurate particle linking events, this analysis was done with very conservative particle linking criteria, corresponding to a particle link range of 5 image frames and a maximum allowed particle displacement of one pixel per image frame. The detected particle motion data was post-processed using custom written Mathematica routines. This post-processing included further linking of particle trajectories by a coincidence search routine in time and space of all trajectories, with a minimal length greater than a cut-off value (here n = 10 to 20 frames) with other trajectories that coincided within space, δr (here set to 8 pixels) but not time. We next calculated the mean squared displacements for each single trajectory, m, that contained n>20 displacements, and for all possible time intervals, nτ, [16]

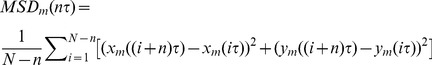
(4)where τ is the acquisition time interval and N is the total number of displacements in a trajectory. The average diffusion coefficient, 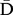, of all trajectories for a given molecule and label was then determined by calculating the average mean squared displacements <MSD(nτ)> at each time interval, n τ, for each individual trajectory, m
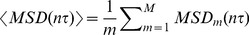
(5)


The average diffusion coefficient was then determined by curve fitting the initial five time points, with fit weights equal to the inverse variance (1/σ^2^), to the expression for free diffusion plus a constant, c,
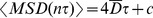
(6)where 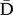 is the average diffusion coefficient.

Furthermore, the mean squared displacement for all single trajectories that contained n>50 frames were analyzed at time intervals, 1≤n≤5, corresponding to a maximum time displacement of approximately 0.2 s respectively, by curve fitting to three simple motion models,

1. Free diffusion,
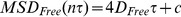
(7)
2. Confined diffusion,
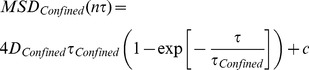
(8)and
3. Mixed transient diffusion
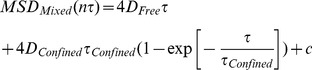
(9)where D_Confined_ is the confined diffusion coefficient, τ_Confined_ is the confinement time, and where the area of the confinement region, L^2^, is given by
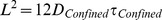
(10)

Having thus fitted each of the experimentally determined single molecule trajectories to all three theoretical models (Eqs. 7–9) for the time interval, 1≤n≤5, we then used F-statistics in order to identify the simplest model (i. e. the model with the least number of free fitting parameters) that described the data. Specifically, this was done by use of the F-test, where we calculated the F-statistic
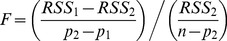
(11)where n is the number of data points, RSS_i_ is the residual sum of squares of model i, and p_i_ is the number of free fitting parameters of model i, and where p_2_>p_1_. We next compared the calculated F-statistics for the individual trajectories to the critical F distribution with (p_2_-p_1_,n-p_2_) degrees of freedom and a significance level of p = 0.05. Fitted values for all trajectories best described as free diffusion are shown as Box- and- Whisker plots (Figures 3e and 4d). In these plots, the box ranges from the first to the third quartiles, and where the statistical median is the center line in the box, while the whiskers extend to the farthest point that are within 3/2 of the interquartile range, and where remaining points are plotted individually as outliers, indicating the presence of heterogeneity among single trajectories.

### Comparison of SPT and kICS Analysis

The percentage difference of the results for the average diffusion coefficient, 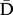, determined by kICS and SPT analysis was calculated by
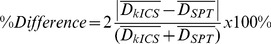
(12)

